# Differential Placental DNA Methylation of *NR3C1* in Extremely Preterm Infants With Poorer Neurological Functioning

**DOI:** 10.3389/fped.2022.876803

**Published:** 2022-06-01

**Authors:** Nienke H. van Dokkum, Sofia Bachini, Rikst Nynke Verkaik-Schakel, Dyvonne H. Baptist, Sahar Salavati, Karianne E. Kraft, Sicco A. Scherjon, Arend F. Bos, Torsten Plösch

**Affiliations:** ^1^Department of Pediatrics, Division of Neonatology, Beatrix Children's Hospital, University Medical Center Groningen, University of Groningen, Groningen, Netherlands; ^2^Department of Obstetrics and Gynaecology, University Medical Center Groningen, University of Groningen, Groningen, Netherlands

**Keywords:** placenta, DNA methylation, stress, prematurity, neurological functioning

## Abstract

**Background:**

Understanding underlying mechanisms of neurodevelopmental impairment following preterm birth may enhance opportunities for targeted interventions. We aimed to assess whether placental DNA methylation of selected genes affected early neurological functioning in preterm infants.

**Methods:**

We included 43 infants, with gestational age <30 weeks and/or birth weight <1,000 g and placental samples at birth. We selected genes based on their associations with several prenatal conditions that may be related to poor neurodevelopmental outcomes. We determined DNA methylation using pyrosequencing, and neurological functioning at 3 months post-term using Prechtl's General Movement Assessment, including the Motor Optimality Score-Revised (MOS-R).

**Results:**

Twenty-four infants had atypical MOS-R, 19 infants had near-optimal MOS-R. We identified differences in average methylation of *NR3C1* (encoding for the glucocorticoid receptor) [3.3% (95%-CI: 2.4%−3.9%) for near-optimal vs. 2.3% (95%-CI: 1.7%−3.0%), *p* = 0.008 for atypical], and at three of the five individual CpG-sites. For *EPO, SLC6A3, TLR4, VEGFA, LEP* and *HSD11B2* we found no differences between the groups.

**Conclusion:**

Hypomethylation of *NR3C1* in placental tissue is associated with poorer neurological functioning at 3 months post-term in extremely preterm infants. Alleviating stress during pregnancy and its impact on preterm infants and their neurodevelopmental outcomes should be further investigated.

## Introduction

Approximately one in 10 infants is born preterm, before 37 completed weeks of gestation (1). Despite substantial improvements in modern neonatal care, preterm infants, especially those born very to extremely preterm, are at higher risk of adverse neurodevelopmental outcomes (2). These neurodevelopmental impairments may include cognitive and motor delays and socio-emotional functioning, both short-term and long-term (3, 4).

The causes leading to neurodevelopmental impairment following preterm birth are multifactorial and not always well understood. One theory is that adverse intrauterine conditions can lead to “fetal programming,” which in turn may have implications for neurodevelopment. Epidemiological and clinical studies report that adverse fetal environments could have major impacts on pathophysiological processes across the lifespan (5), which supports this theory. A mechanism through which fetal programming can occur regards DNA methylation, which is an epigenetic alteration that regulates gene expression. Several adverse intrauterine conditions may play a direct or indirect role in neurodevelopmental impairment following preterm birth, including inflammation, hypoxia, reduced supply of macro- and micronutrients in fetal growth restriction with resulting altered angiogenesis, delayed neuronal growth, and an altered stress response. All these conditions may affect DNA methylation of associated genes.

Several genes of interest that may impact neurodevelopmental impairment include the genes encoding for erythropoietin (*EPO*), the sodium-dependent dopamine transporter (solute carrier family 6 member 3, *SLC6A3*), toll-like receptor 4 (*TLR4*), vascular endothelial growth factor A (*VEGFA*), leptin (*LEP)*, the glucocorticoid receptor (nuclear receptor subfamily 3 group C member 1, *NR3C1*) and hydroxysteroid 11-beta dehydrogenase 2 (*HSD11B2*). *EPO* is mainly associated with the proliferation, maturation, and differentiation of red blood cells, but has also been proven important in the direct development, maintenance, protection, and repair of the central nervous system (6). *SLC6A3* encodes for the dopamine transporter, with dopamine being an important neurotransmitter for the central nervous system. *SLC6A3* plays a role in the dopamine re-uptake pathway and has been identified as a key gene in neurodevelopment (7). *TLR4*, belonging to the family of pattern recognition receptors, is involved in immunomodulation. It binds to bacterial lipopolysaccharides, which triggers the intracellular signaling pathway of innate immune response activation, and production of pro-inflammatory cytokines (8). In the case of preterm birth, this pathway is often upregulated, which signals intrauterine inflammation (8). *VEGFA* plays an important role in angiogenesis, which is required for endothelial cell proliferation and growth (9), and may be related to hypoxic states during pregnancy due to placental insufficiency, affecting preterm growth and development. *LEP* is a crucial metabolic hormone for body weight regulation, placental and fetal growth, that is highly expressed in the placenta (10). *LEP* is also important for angiogenesis and brain development in fetuses (10). *NR3C1* encodes for the glucocorticoid receptor. *HSD11B2* is the enzyme entailing the barrier function of the placenta, responsible for limiting the amount of circulating cortisol that is passed to the fetus, by converting cortisol into its inactive metabolites. High fetal exposure to glucocorticoids, such as cortisol or the “stress hormone”, have been implicated in altered neuroendocrine environment often associated with poor neurological outcomes (11, 12). Together, these genes may provide insight into which processes affect neurodevelopmental outcomes following preterm birth.

A method to reliably assess early neurological functioning is by assessing the early motor repertoire of infants. At 9–16 weeks post-term, the early motor repertoire includes fidgety movements (FMs), small circular movements visible in all joints (13), as well as several other movement and postural patterns, which together form the basis for the motor optimality score – revised (MOS-R) (14). The early motor repertoire, including the MOS-R, is reported to be predictive of cerebral palsy (CP). The absence of FMs predicts CP with a sensitivity of 98% and a specificity of 91% respectively (13). Additionally, for other areas of neurodevelopment, such as neurological deficits, intelligence, and cognition, evidence is accumulating that this non-invasive tool is predictive of even minor issues as well, from childhood up to and including adulthood (15–17).

Understanding the underlying mechanisms of neurodevelopmental impairment following preterm birth may enhance opportunities for targeted early interventions. Therefore, our aim was to assess whether placental DNA methylation of the aforementioned genes affected early neurological functioning in preterm infants born before 30 weeks of gestation.

## Materials and Methods

### Study Population

We screened the first 70 infants with a gestational age of <30 weeks and/or a birth weight of <1,000 g that participated in the prospective cohort study NeoLifeS for eligibility in the current study. For this hypothesis-generating study, a feasible sample size of about 40 infants was determined, based on a previous study from our group (18). Infants were excluded for the current study if they had missing placenta samples (*n* = 6) or missing placental examinations, which were performed by a pathologist (*n* = 19). Two additional infants were excluded because of extremely poor quality of the placental samples. Our final sample therefore consisted of 43 infants. The NeoLifeS cohort study was approved by the Institutional Review Board of the University Medical Center Groningen (METc 2013/262). Written informed consent was obtained from all parents.

### Selection of Genes and Primer Design

We selected relevant genes based on their associations with several prenatal conditions that may be related to poor neurodevelopmental outcomes. The genomic target region we chose (Table 1) was based on existing literature. All primers for the target regions were designed using the Pyromark Assay Design software (Qiagen). We preferably selected CpG-rich areas in the promotor part of the gene because of their putative regulatory function on transcriptional activity. For *EPO*, the analyzed promotor region includes the binding site for the hypoxia inducible factor (HIF) complex, that has been reported to repress transcription through hypermethylation in cancer (19). *SLC6A3* has been identified to be differentially methylated in preterm newborn by Arpón et al. (7). Our assay was designed to include their position Cg00997378. For *TLR4* the selected area included a binding site for Sp-1 and regulatory factor X1, a transcription suppressor, strongly associated with *TLR4* expression (20, 21). For *VEGFA* we included an area with a hypoxia inducible factor complex site where hypomethylation is present in placental tissue after preeclampsia (22). Our investigated *LEP* region overlaps with the promotor region, that is reported to be regulated by methylation (23). For *NR3C1*, we analyzed the locus also identified by Giarraputo and colleagues, also using the same reverse primer (24). Lastly, our *HSD11B2* assay was designed to include several CpG positions defined in literature, especially those found by Marsit et al. (25).

**Table 1:** PCR forward and reverse primer sequences accompanied by sequencing primer and the sequence to analyze, and their genomic region.

**Gene**	**Primers**	**Sequence to analyze**	**Genomic region**
*EPO*	F:5′-GGGGGTAGGGGTTGTTATTTGTATG-3′ R:5′-Biotin-CCCAAACCTCCTACCCCTACTCTAACC-3′ S 5′-GGGTTGTTATTTGTATGTG-3′	TGYGTGYGYGGGTGGGGGTG GGGGAGAGGTTGTGTGYGTG AGGGGTYGTTAGGGGTAGGG GTTATTYGGGGTTAGAGTAG GGGTAGGA	Chromosome 7: 100720774–100720822
*SLC6A3*	F:AGTTATATTTATTTAGGGTAGGTGGTATT R:5′-Biotin-AATTTCCCCAATTACCCTACTAACCC S:AGGGTAGGTGGTATTAT	TTYGYGTGAGAGAGTTGGGY GGAGGATGGATAGGGTTTTA TYGYGGGAATTAGTTTTTGG GTTAGTAGG	Chromosome 5: 1443000–1448000
*TLR4*	F:5′GTTGAGGTTTATTTTTAGTTTTGTATGTG3′ R: 5′-Biotin-AACCTCATTCTACCTTACATACC3′ S:5′GTGAGTTTTTTTATAAGAAGGG 3′	GYGGGTTAAA TTGTGTTTTG TAAAAATTTA TATATYGAAG TTTTAATTTT TTTATTTTAG A	Chromosome 9 117703726–117703786
*VEGFA*	F:5′-GGGAGTAGGAAAGTGAGGT-3′ R:5′-Biotin-TTCCCCTACCCCCTTCAATAT-3′ S:5′-AGTAGGAAAGTGAGGTTA-3′	YGTGYGGATA GGGTTTGAGA GTYGTTTTTT TTTTGTTAGG AATATTGA	Chromosome 6: 43769854–43769901
*LEP*	F: 5′-GGTGTATATTGAGGGTTTAGGGTTAGTA-3′ R: 5′-Biotin-CCATACCTACCCCCCCCTCTTATAAC-3′ S1: 5′- GGTTTAGGGTTAGTAGT -3′ S2: 5′- GGGAGTTGGAGTTAGAAATG -3′	S1:YGTTYGGTAYGTYGTTATTTTGAGGGGYGGGGY GGGAGTTGGYGTTAGAAATG S2:YGTYGGGGTTTGYGGGGTAGTTGYGTAAGTTGTGA TYGGGTYGTTATAAGAGGGGYGGGTAGGTATGGAGTTT	Chromosome 7: 128241151–128241276
*NR3C1*	F:5′- AGTTTTAGAGTGGGTTTGGAG -3′ R:5′- Biotin- CCCCCAACTCCCCAAAAA-3′ S:5′-GAGTGGGTTTGGAGT−3′	YGYGGAGTTGGGYGGGGGYG G GAAGGAGGTAGYGAGAAAAGAAATTGGAGAAATT	Chromosome 5: 143403500–143405000
*HSD11B2*	F:5′-Biotin-GGGTGTGTGAGTTAGGGATTT-3′ R:5′-ACATCCCCATACCCTTTACTAATC−3′ S:5′-AACCAACCCATACTCACC−3′	CRATCTCCRCTACCRCTACCRCTACCRC CCRCRCCCRTACACRCRCCRCACTCCCAC	Chromosome 16: 67427000–67432000

*F, forward primer; R, reversed primer; S ,sequence primer; S1, sequence 1; S2, sequence 2*.

### Quantification of Methylation and Pyrosequencing

Paraffin-embedded placenta samples of the fetal side were provided by the pathology department, after standard assessment had been performed. Five cut cores with a diameter of five μm per core were used per placental sample to isolate DNA. DNA isolation was performed using the QIAamp DNA Formalin-Fixed, Paraffin-Embedded Tissue Kit (Qiagen). Quality and concentration of the isolated DNA was assessed using the NanoDrop®ND-1000 spectrophometer (Thermo Fisher Scientific, Waltham, MA). Obtained concentrations ranged between 121.1 and 482.4 ng/μl. Afterwards, DNA was bisulfite converted, using the EZ methylation Gold-Kit (Zymo Research, Irvine, CA), according to the supplier's protocol. In total, 500 ng of DNA sample was bisulfite converted for subsequent analysis. For polymerase chain reaction (PCR) amplification, we used a master mix of 12.5 μl HotStarTaq DNA Polymerase, 10.5 μl sterile water, a 1 μl mix of forward and reverse primer and a 1 μl bisulfite template, according to the manufacturer's instructions. We included a negative control to check for contamination. Cycling conditions for PCR were the same for all assays, except for *HSD11B2*: 95°C for 15 min, 45 cycles of 94°C for 30 s, 58°C (*HSD11B2* 56°C) for 30 s, 72°C for 30 s, followed by a final step of 72°C for 7 min. Afterwards, a DNA ladder and 3–5 μl of each PCR product was loaded and run on 2% agarose gel with ethidium bromide staining to visualize presence or absence of PCR products and contamination. Next, pyrosequencing was performed using the PyroMarkQ24 (Qiagen). Methylation levels were analyzed with associated software. Each of the CpG sites was quality control checked and the percentage of DNA methylation at individual CpG sites was calculated. Additional information on the pyrosequencing method used and two representative examples of the sequencing results are provided in Supplementary Figure 1.

### Early Neurological Functioning

Infants were videotaped for approximately 10 min at 3 months post-term to assess their spontaneous movement repertoire. During the recording the infants were in active wakefulness, partly dressed, and in supine position. All video recordings were assessed according to Prechtl's GMA (13). Each video recording was assessed by two trained assessors, who were blinded to the infant's medical history. In case of disagreement, a senior assessor (AFB), also a licensed GM tutor, was consulted. In previous studies, the inter-scorer agreement for the MOS-R was reported to be good to excellent with Cohen's Kappa statistics between 0.75 and 0.91 (26). First, the infants' FMs were classified as either normal or aberrant (meaning abnormal or absent). Second, the MOS-R was determined. The MOS-R consists of five categories: FMs, observed movement patterns, age-adequacy of the movement repertoire, observed postural patterns, and movement character. Points awarded according to the manual are 4 for normal, 2 for reduced and 1 for absent. For FMs, scoring is slightly different, with 12 points awarded for normal FMs, 4 points for abnormal FMs, and 1 point for absent FMs. After individual categories are scored, the sum of the categories forms the MOS-R, ranging from 5 to 28 points. A score between 25 and 28 was considered near-optimal, whereas a score below 25 was considered atypical.

### Statistical Analyses

First, we present demographic data using descriptive statistics. Distribution of data was assessed using visual inspection of Q–Q plots, as well as a Shapiro–Wilk test. Second, we tested differences in average DNA methylation of the selected genes between infants with near-optimal MOS-R and atypical MOS-R using independent samples *t*-tests or Mann–Whitney-*U* tests as appropriate. For genes that were significantly different between the two groups, we also tested differences in individual CpG sites between infants with near-optimal MOS-R and atypical MOS-R using independent samples *t*-tests or Mann-Whitney *U*-tests as appropriate. When a statistically significant result was found, we then performed a univariable and multivariable logistic regression analysis, in which we adjusted for sex and mode of delivery. Analyses were performed using SPSS for Windows, version 28.0 (IBM Corporation, NY, USA). A *p*-value <0.05 was considered statistically significant.

## Results

### Characteristics of the Study Population

In this study, we included 43 neonates. We present all participant characteristics in Table 2. Characteristics did not differ between infants who had an atypical MOS-R of <25 and infants with a near-optimal MOS-R of 25–28. None of the infants suffered from severe intracranial hemorrhages (grade 3 or 4), cystic periventricular leukomalacia, or cerebellar hemorhages. During the pyrosequencing analysis, the quality of some samples was too low for methylation to be measured, which resulted in unequal sample sizes for each gene. The MOS-R ranged from 12 to 28, with 24 infants scoring below 25.

**Table 2:** Participant characteristics (*N* = 43).

	**MOS-R <25** **(*n* = 24)**	**MOS-R 25–28,** **(*n* = 19)**	***P*** **-value**
Gestational age (weeks)	27.6 ±1.52	27.5 ±1.50	0.85^T^
Birth weight (g)	1,023.2 ±216.4	986.6 ±279.1	0.63^T^
Small for gestational age	3 (12.5)	2 (10.5)	0.84^F^
Male sex	14 (58.3)	8 (42.1)	0.36^F^
Multiple pregnancy	2 (8.3)	4 (21.1)	0.38^F^
C-section delivery	10 (41.7)	12 (63.2)	0.86^X^
Prenatal steroids	15 (62.5)	15 (78.9)	0.24^X^
Apgar score	8 (7–8)	8 (7–9)	0.34^U^
NICU stay (days)	15.0 (5.0–29.0)	16.5 (8.3–23.8)	0.57^U^
Postnatal steroids	5 (20.8)	3 (15.7)	1.00^F^
Mechanical ventilation (days)	6 (1–11)	2 (1–19)	0.83^U^
NEC	3 (12.5)	5 (26.3)	0.43^F^
Sepsis	7 (29.2)	3 (15.7)	0.47^F^
PDA	12 (50.0)	10 (52.6)	1.00^F^
Genes present			
*EPO*	20 (83.3)	18 (94.7)	0.36^F^
*SLC6A3*	19 (79.2)	18 (94.7)	0.21^F^
*TLR4*	20 (83.3)	16 (84.2)	1.00^F^
*VEGFA*	23 (94.8)	16 (84.2)	0.31^F^
*LEP*	20 (83.3)	16 (84.2)	1.00^F^
*NR3C1*	18 (75.0)	17 (89.5)	0.27^F^
*HSD11B2*	14 (48.3)	12 (63.2)	0.75^F^

*Characteristics given as mean ± SD, median (P25–P75), n (%) according to their distribution*.

*MOS-R, Motor Optimality Score-Revised; NICU, Neonatal Intensive Care Unit; NEC, Necrotizing Enterocolitis defined as Bell stage >2A; PDA, Persistent Ductus Arteriosus treated with ibuprofen or clip. Sepsis was blood culture proven. Small for gestational age was defined as a birth weight below the 10th percentile on Dutch growth charts*.

U *Mann–Whitney U-Test*,

T *Students T-test*,

X *Chi-square Test*,

F *Fisher's Exact Test*.

### Average DNA Methylation of Selected Genes

We present the average methylation for each of the included genes in Table 3. We found a statistically significant difference for *NR3C1* between the two groups of 3.3% (95% CI: 2.4%−3.9%) vs. 2.3% (95% CI: 1.7%−3.0%), *p* = 0.008. In univariable logistic regression analysis the odds ratio for an atypical MOS-R with each percent increasing methylation in the *NR3C1* gene was 3.23 (95% confidence interval 1.26–8.28, *p* = 0.015). After adjustment for sex and mode of delivery, results remained statistically significant (odds ratio 3.36, 95% confidence interval 1.21–9.37, *p* = 0.020). For *EPO, SLC6A3, TLR4, VEGFA, LEP* and *HSD11B2* we did not find statistically significant differences between the two groups.

**Table 3:** Average methylation of the selected genes.

**Gene**	***N***	**Median methylation (IQR) MOS-R <25**	**Median methylation (IQR) MOS-R 25–28**	***P*** **-value**
*EPO*	38	2.4 (1.7–2.5)	2.4 (2.1–2.8)	0.34
*SLC6A3*	37	45.5 (39.2–51.9)	44.8 (40.9–51.3)	0.84
*TLR4*	36	40.8 (37.8–47.8)	41.1 (35.2–48.5)	0.74
*VEGFA*	39	3.2 (3.0–3.5)	3.3 (3.0–4.0)	0.86
*LEP*	36	19.6 (13.9–27.4)	20.1 (16.0–26.0)	0.92
*NR3C1*	35	2.3 (1.7–3.0)	3.3 (2.4–3.9)	0.008
*HSD11B2*	26	3.1 (3.1–4.9)	2.1 (1.5–2.9)	0.10

*N, number of infants; IQR, interquartile range; MOS-R, motor optimality score*.

### Methylation of Individual NR3C1 CpG-Sites

**Figure 1:**
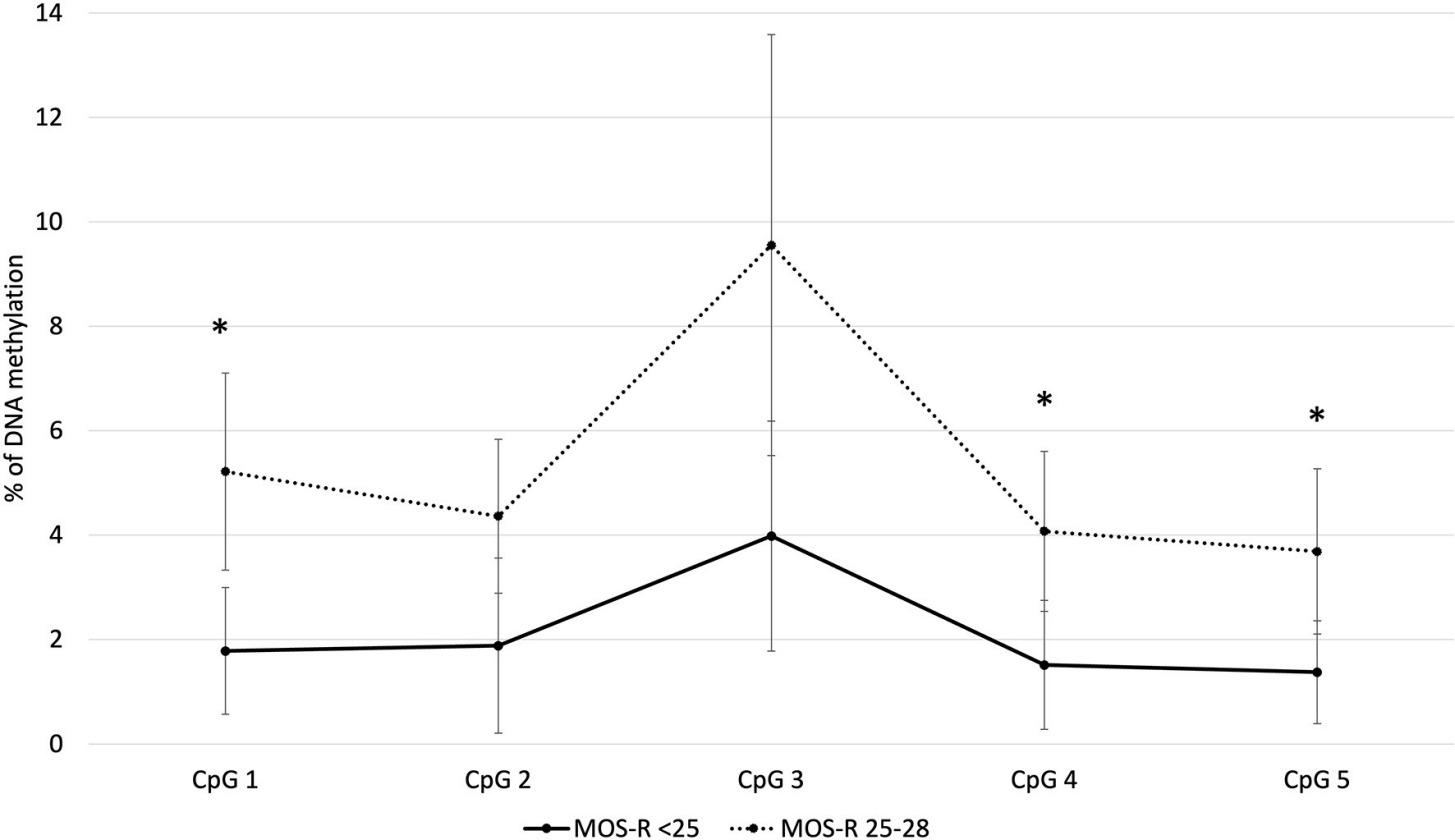
Average methylation for the *NR3C1* gene individual CpG sites. ^*^p< 0.05. MOS-R, Motor Optimality Score-Revised.

Of the individual CpG sites analyzed within the *NR3C1* gene (Figure 1), CpG 1, CpG 4 and CpG 5 showed statistically significantly higher methylation for infants who had a near-optimal MOS-R of 25–28. At CpG 1, median methylation for infants with an atypical MOS-R was 1.8%, compared with 2.9% for infants with a near-optimal MOS-R (U = 291.5, *z* = 2.9, *p* = 0.003). At CpG 4, median values were 1.4 vs. 2.3% (*U* = 255, *z* = 2.2, *p* = 0.024). At CpG 5 median values were 1.3 vs. 2.1% (*U* = 248, *z* = 2.0, *p* = 0.048). CpG 2 and CpG 3 also showed higher methylation in infants with a near-optimal MOS-R, but without statistical significance.

## Discussion

This study aimed to assess whether DNA methylation of seven selected genes, potentially associated with early neurological functioning, affected this outcome in extremely preterm infants. We demonstrated that infants with an atypical MOS-R had lower DNA methylation levels in the *NR3C1* gene overall and in three of the five individual CpG sites, compared with infants with a near-optimal MOS-R. For the other six genes, *EPO, SLC6A3, TLR4, VEGFA, LEP* and *HSD11B2*, we found no differences in DNA methylation levels between the two groups.

We found placental hypomethylation of three out of the five individual CpG sites in the *NR3C1* gene for infants with poorer neurological functioning at 3 months post-term. Our results are in line with those of others, who also reported placental hypomethylation of *NR3C1* to be associated with neurobehavior in fullterm infants, showing poorer regulatory behavior in infancy (27, 28). In contrast, Sheinkopf and colleagues report that higher mean methylation of *NR3C1* associates with poorer regulation reflected in fullterm cry acoustics (29). Our findings may be explained by the influence of maternal cortisol on the development of the stress response as coordinated by the hypothalamic-pituitary-adrenal (HPA) axis (30). Methylation of *NR3C1* has been inversely associated with expression (30), which would suggest that infants with high maternal stress during pregnancy and resulting decreased methylation are potentially exposed to higher cortisol levels in utero. Exposure to higher cortisol levels may then account for adverse infant neurodevelopment. In a systematic review by Zijlmans and colleagues, the effects of prolonged in utero cortisol exposure are noted to be physical, i.e., lower birth weight or being born small-for-gestational age, as well as developmental, in infant motor and cognitive functioning (31). Additionally, in their review, Waffarn and colleagues report effects of in utero exposure to cortisol on the development of the developing HPA axis (32). This may have implications for future treatment in the NICU as well, because the HPA axis is necessary to cope with stressful stimuli. Without this adaptive mechanism properly developed, infants may not be able to regulate stress, and this may in turn hamper neurodevelopment, evidenced by a wide range of effects of neonatal stress exposure (33). The hypothesis that exposure to stress is related to hypomethylation of the *NR3C1* gene is also strengthened by postnatal findings in preterm infants showing that increased stress exposure during NICU stay leads to hypomethylation of the *NR3C1* gene (24). Therefore, the clinical relevance of *NR3C1* methylation may be to identify infants at risk of a variety of neurodevelopmental impairments already very early.

To our surprise, we did not find any differences between the groups in the *HSD11B2* gene. Previous studies reported that the *HSD11B2* and *NR3C1* genes are interrelated, with hypomethylation in *NR3C1* being correlated with hypermethylation in *HSD11B2* (12, 34). In our study, we did observe hypermethylation in the *HSD11B2* gene for infants with poorer neurological outcome even though this did not reach statistical significance, which aligns with these findings. We believe that our findings could be explained by a lack of power to detect differences in methylation in *HSD11B2*, because of a smaller sample size for this gene. Future studies should include both genes, as well as other genes in the glucocorticoid receptor signaling pathway, and focus on the interplay of both genes in the neurodevelopmental pathway.

We also did not find any differences between the groups for the other genes, i.e., *EPO, SLC6A3, TLR4, VEGFA* and *LEP*. Research on these genes and their interplay with neurodevelopment is limited. *EPO* and *TLR4* have been suggested as important genes in neurodevelopment, but studies associating differential methylation in placenta or other tissues with neurodevelopmental outcomes, have, to our knowledge, not been published. For *SLC6A3*, Arpón et al. (7) performed a study that showed a potential role for this gene in neurodevelopment when examining differences between methylation profiles in preterm and fullterm children. This gene has mainly been associated with attention-deficit/hyperactivity disorder (7), but entailing features may not be evident in the MOS-R yet. Methylation of *VEGFA* has been associated with performance intelligence quotient, in infants with brain sparing due to fetal growth restriction (35). Our study included only five infants born small for gestational age, thereby limiting power to detect such associations. Finally, for *LEP*, one study by Lesseur et al. (30), reports that placental hypermethylation is associated with poorer neurobehavior expressed as lethargy and hypotonicity, in males only. Both our groups entailed equal numbers of males and females, which may have masked such an effect, or we simply may have lacked power to detect differences.

The role of the placenta as a fundamental link between the intrauterine environment and early neurodevelopmental outcomes has become more and more evident. Nugent and Bale (36) even define this link as the “diplomat for maternal-fetal relations.” The intrauterine environment is influenced by both gestational age and environmental factors, that may trigger placental adaptations which reflect in the fetal development (36). Our study focused on key processes that are thought to be relevant in the interaction between placental adaptations and fetal development, including central nervous system development, inflammation, angiogenesis, metabolic processes, and the stress response. Of these processes, the intrauterine stress response seems to be the most influential for atypical neurodevelopment among extremely preterm infants.

### Strengths and Limitations

To the best of our knowledge, our study is the first study investigating the role of placental DNA methylation of several neurodevelopmentally important genes and their association with early neurological functioning in a group of preterm infants born before 30 weeks' gestation. Other studies focused on differences between preterm and fullterm birth, while ours distinguished between preterm infants with and without (minor) neurological impairments. Based on their placental DNA methylation profile, we studied potential pathophysiological pathways that may be responsible for the neurological impairments, to early identify infants that could benefit from intervention. Additionally, the technique of general movements assessment is a very reliable assessment that is highly predictive for later-life outcomes (13, 37–40). We also acknowledge our limitations. First, we used paraffin-embedded placental tissue, with less optimal quality than fresh placenta material, making DNA isolation more difficult. In future studies, fresh placental tissue samples may be more optimal. Second, we did not investigate gene expression and/or hormone levels, which hampers us to draw definitive conclusions on the impact of the observed DNA methylation patterns. We purposely chose to describe our observation without postulating a direct clinical consequence, because of three main reasons. First, DNA methylation is not the only factor determining gene expression, as also the presence of specific transcription factors is required. Second, expression may be tissue specific, and in this study, we use the placenta as a surrogate for a general pattern, in this case the brain. Third, we measured changes in a homogenate of placenta tissue, meaning that methylation changes in a subpopulation of cells might be much higher. In theory, single-cell analysis methods or staining methods would be suitable to get more insights, if no post-transcriptional regulation takes place, but these methods may not be sensitive enough to detect differences equivalent to the 1% methylation difference we observed. Still, an inverse relation between methylation and expression has previously been described in the *NR3C1* gene (30). As a next limitation, our study included a limited number of only 43 infants. Larger prospective studies are warranted to confirm our results. Those studies may also include gene expression analyses to further elucidate the relation between DNA methylation and expression. A final limitation regards multiple testing. However, because of the hypothesis generating nature of this study, we chose not to correct for multiple tests performed, thereby acknowledging that some of our results may be chance findings. Because of the small sample size and hypothesis generating nature of this study, we purposely chose not to perform genome-wide arrays.

## Conclusion

In conclusion, in our study of 43 extremely preterm infants, the stress response during pregnancy and related hypomethylation in placental tissue is associated with poorer neurological functioning at 3 months post-term. We did not identify evidence of such associations for other processes associated with preterm birth. Alleviating stress during pregnancy and its impact on preterm infants and their neurodevelopmental outcomes should be further investigated.

## Data Availability Statement

The raw data supporting the conclusions of this article will be made available by the authors, without undue reservation.

## Ethics Statement

The studies involving human participants were reviewed and approved by Institutional Review Board of the University Medical Center Groningen (METc 2013/262). Written informed consent to participate in this study was provided by the participants' legal guardian/next of kin.

## Author Contributions

SB, SS, AB, and TP were involved in the design of the study. SB, RV-S, and TP performed the laboratory analyses. DB, SS, KK, and AB performed the General Movement Assessment. NvD performed the statistical analyses and drafted the first and final version of the manuscript. All authors revised the manuscript critically for important intellectual content and approved the final manuscript for publication.

## Funding

NvD was financially supported by a grant from the Junior Scientific Masterclass of the University of Groningen. The financers have had no role in any stage of the project, including the decision to submit the manuscript.

## Conflict of Interest

The authors declare that the research was conducted in the absence of any commercial or financial relationships that could be construed as a potential conflict of interest.

## Publisher's Note

All claims expressed in this article are solely those of the authors and do not necessarily represent those of their affiliated organizations, or those of the publisher, the editors and the reviewers. Any product that may be evaluated in this article, or claim that may be made by its manufacturer, is not guaranteed or endorsed by the publisher.

## Supplementary Material

Supplementary Figure 1 Representative Pyrograms. We here show two representative pyrograms for the NR3C1 sequence. **(A)** Sample 32, MOS26, birth weight 1,180 g **(B)** sample 39, MOS 22, birth weight 1,200 g. Blue boxes and the tables give the methylation percentage as measured by the Pyromark Q24 system. The Pyromark Q24 system measures light signals which are linearly correlated to the incorporation of the specific nucleotides into the sequence. In short, the specific nucleotides are added in their predefined order. This sequence is given in the figure (“Sequence to analyze”). At the putative methylation sites (marked as “Y” in the sequence-to-analyze), the system adds both C and T after each other, which are equivalent to methylated and non-methylated CpG positions, respectively. The software subsequently calculates the methylation percentage at those positions as the ratio between the two signals. Non-CpG cytosines and thymidines are used for calibration purposes and as quality control. The blue boxes in the pyrogram are the imputed methylation levels of the light-blue shaded position. The blue color of the boxes also indicates that the signal here fulfills the internal quality criteria (otherwise it would be yellow or red). The yellow-shaded position serves as an internal negative control, as the called “C” does not occur in the genomic sequence.
