# Supplementary figures and images for: Differential Placental DNA Methylation of NR3C1 in Extremely Preterm Infants With Poorer Neurological Functioning
Source: Front Pediatr. 2022 Jun 1;10:876803. doi: 10.3389/fped.2022.876803 (PMC9198301; doi:10.3389/fped.2022.876803)

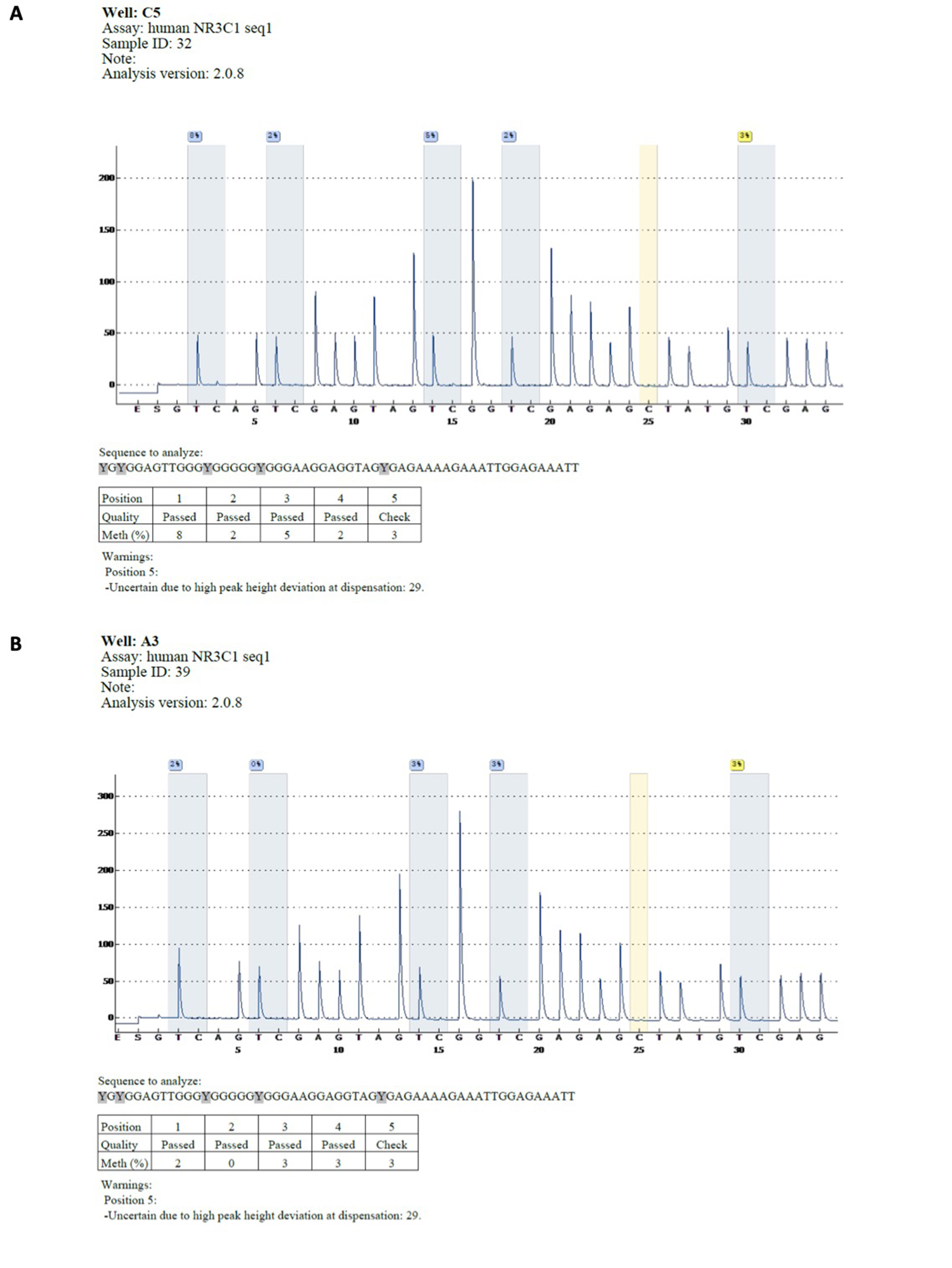

Supplement: Supplementary Figure 1 — Representative Pyrograms. We here show two representative pyrograms for the NR3C1 sequence. (A) Sample 32, MOS26, birth weight 1,180 g (B) sample 39, MOS 22, birth weight 1,200 g. Blue boxes and the tables give the methylation percentage as measured by the Pyromark Q24 system. The Pyromark Q24 system measures light signals which are linearly correlated to the incorporation of the specific nucleotides into the sequence. In short, the specific nucleotides are added in their predefined order. This sequence is given in the figure (“Sequence to analyze”). At the putative methylation sites (marked as “Y” in the sequence-to-analyze), the system adds both C and T after each other, which are equivalent to methylated and non-methylated CpG positions, respectively. The software subsequently calculates the methylation percentage at those positions as the ratio between the two signals. Non-CpG cytosines and thymidines are used for calibration purposes and as quality control. The blue boxes in the pyrogram are the imputed methylation levels of the light-blue shaded position. The blue color of the boxes also indicates that the signal here fulfills the internal quality criteria (otherwise it would be yellow or red). The yellow-shaded position serves as an internal negative control, as the called “C” does not occur in the genomic sequence. [file Image_1.JPEG]
